# Supplementary material for: Drones and digital adherence monitoring for community-based tuberculosis control in remote Madagascar: A cost-effectiveness analysis
Source: PLoS One. 2020 Jul 7;15(7):e0235572. doi: 10.1371/journal.pone.0235572 (PMC7340291; doi:10.1371/journal.pone.0235572)
Supplement: Attachment — Submitted filename: DrOTS_CEA_ResponseToReviewers_V1.docx [file pone.0235572.s001.docx]

**Manuscript Number:** PONE-D-20-10161

**Manuscript Title:**	Drones and digital adherence monitoring for community-based tuberculosis control in remote Madagascar: a cost-effectiveness analysis

**Journal:**	PLOS ONE

Montreal May 16^th^

Dear Frederick Quinn, editor

We wish to. thank you and the two external reviewers for reviewing our paper in detail and providing useful comments and suggestions.

We have carefully reworked our manuscript in light of your reports. Below, please find our point-by-point response, clearly indicating how and where in the manuscript (line numbers) changes have been made. Altered text has been highlighted throughout the manuscript using a yellow marker to assist you in readily reviewing our changes made.

We feel that our revised manuscript has gained in clarity and quality, and hence, we hope that it is now suitable for publication in *PLOS One.*

Yours sincerely,

Dr. Simon Grandjean Lapierre MD MSc FRCPC

On behalf of all the authors

EDITOR’S COMMENTS

COMMENT #1 – Please ensure that your manuscript meets PLOS ONE's style requirements, including those for file naming.

ANSWER #1 – The formatting was adjusted as requested in the reviewed version. (See highlights throughout the manuscript)

COMMENT #2 – We note that you have indicated that data from this study are available upon request. PLOS only allows data to be available upon request if there are legal or ethical restrictions on sharing data publicly. For information on unacceptable data access restrictions, please see <http://journals.plos.org/plosone/s/data-availability#loc-unacceptable-data-access-restrictions>.

AMSWER #2 – There are no ethical or legal restrictions on sharing this study’s data set. This is a modeling study. The primary data for this study is as described in the “Decision analysis model” and “Costs” sections and in “Table 1” and “Table 2”. It includes Madagascar’s National TB program data and TB disease pathophysiology information which is already in the public domain and appropriately referenced. It also includes. Madagascar’s TB clinics operation cost and multiple bills and financial statements from the DrOTS project and implementing institutions. We believe the methods and detailed data sources provided in the manuscript text allows for reproduction of our results. If further proof of data is needed (e.g. bill of drone purchase, bill of electricity for drone charging) we are committed to providing this information to whom would be interested but feel it is not a dataset which would be helpful to the scientific community on a public repository. Please do update our Data Availability statement on our behalf to reflect this information.

COMMENT #3 – Thank you for stating the following in the Competing Interest section: The authors have declared that no competing interests exist.

ANSWER #3 – This was added to the manuscript. (Line 597)

COMMENT #4 – We note that one or more of the authors are employed by a commercial company: ValBio Research Center. Please provide an amended Funding Statement.

ANSWER #4 – ValBio Research Center is not a commercial company. It is a Research Campus owned by Stonybrook University and operating in Madagascar under a Non-Profit Organization legal status. It is part of the academic institution. ValBio Research is therefore not a sponsor or beneficiary of this study. Hence, we did not modify any of the authors’ contribution or Competing Interests Statement. Regarding the Funder (Stop TB Partnership), we have amended the Funding Statement as suggested. The Funding Statement and Competing Interests Statement should now read as follows. We thank the editorial team for making those changes on the online submission form on our behalf. (Line 605)

# *Competing interest*

*The authors have declared that no competing interest exist.*

# *Financial disclosure*

*This work was supported by the Stop TB Partnership’s TB REACH wave 5 award to NR and PMS (http://www.stoptb.org/global/awards/tbreach/wave5.asp). The Stop TB Partnership is funded by the Government of Canada and the Bill and Melinda Gates Foundation.* *AMK is supported by the Rudolf Geigy Foundation, Swiss Tropical and Public Health Institute, Basel, Switzerland. SGL is supported by the Canadian Association for Microbiology and Infectious Diseases. The funder did not provide salary support for principal investigators but did provide salary support for research staff including a co-author on this manuscript (AA). The specific roles of the authors are articulated in the ‘author contributions’ section.” No funders or sponsors played any role in study design, data collection and analysis, decision to publish or preparation of the manuscript.*

REVIEWER 1 COMMENTS

COMMENT #1 – How to deal with the retreatment TB patients, whose treatment course should be 8 months according to WHO guidelines?

ANSWER #1 – Indeed, in this modeling study, we focused on first episodes of drug-susceptible pulmonary TB. To adapt the DrOTS model to different case scenarios including re-treatment cases but also, pediatric TB, extrapulmonary TB and Drug-Resistant TB, would require the construction of different models which would be informed by additional primary collected data. We acknowledge this limitation in the discussion, and we have modified the text to better reflect the additional challenge raised by the reviewer. (Line 554)

COMMENT #2 – The difficulties and challenged encountered during implementing DrOTS should be discussed in detail in order to help others to better understand the innovative intervention, such as how to make sure that the drones could deliver medication to the right patient?

ANSWER #2 – We have provided additional details in the “Modeled strategies / DrOTS” sub-section of the manuscript. (Line 153)

COMMENT #3 – It seems that TB patients under DrOTS will not visit outpatient clinic and see the doctor, so how to do when there is side effect and how to evaluate the treatment outcome for smear-negative without taking chest X-ray examination?
ANSWER #3 – Treatment outcomes for smear-positive cases are assessed with control smears as described in the manuscript. In the decision analysis tree, enrollment in the DOTS or DrOTS arms is based on microbiology testing (either smear or GeneXpert). If not recognized upon first testing, patients are expected to seek medical attention again later in disease course and potentially be diagnosed at this time. This model does not account for smear-negative patients not being diagnosed upon two consecutive testing. As it is the case for pediatric TB, drug resistant TB or extrapulmonary TB, DrOTS does not currently allow for comprehensive management of all TB forms in a de-centralized approach and support from local health centers is still required for certain patients. Although those patients are equally accounted for in both modeled strategies, we do not believe that this impacts our conclusions in terms of ICER or absolute cost per additional TB patient diagnosed.

REVIEWER 2 COMMENTS
COMMENT #2 – The manuscript is well written, comprehensive data analyses are done and clearly presented. The conclusion are meaningful. The findings of the study address a very important and novel area of research where evidence is still very limited. The findings are very informative and will be very useful for improving health systems in Sub-Saharan Africa.

ANSWER #2 – We thank reviewer 2 for seeing the added value of our study to the currently available literature.
